# Flanking sequence context-dependent transcription factor binding in early *Drosophila* development

**DOI:** 10.1186/1471-2105-14-298

**Published:** 2013-10-04

**Authors:** Jessica L Stringham, Adam S Brown, Robert A Drewell, Jacqueline M Dresch

**Affiliations:** 1 Computer Science Department, Harvey Mudd College, 301 Platt Boulevard, Claremont, CA 91711, USA; 2 Biology Department, Harvey Mudd College, 301 Platt Boulevard, Claremont, CA 91711, USA; 3 Department of Biological Sciences, Mount Holyoke College, South Hadley, MA 01705, USA; 4 Department of Biology, Amherst College, Amherst, MA 01002, USA; 5 Mathematics Department, Harvey Mudd College, 301 Platt Boulevard, Claremont, CA 91711, USA; 6 Department of Mathematics, Amherst College, Amherst, MA 01002, USA

**Keywords:** Transcription factor, Binding site, Position weight matrix, Enhancer, *Cis*-regulatory module, *Drosophila*

## Abstract

**Background:**

Gene expression in the *Drosophila* embryo is controlled by functional interactions between a large network of protein transcription factors (TFs) and specific sequences in DNA *cis*-regulatory modules (CRMs). The binding site sequences for any TF can be experimentally determined and represented in a position weight matrix (PWM). PWMs can then be used to predict the location of TF binding sites in other regions of the genome, although there are limitations to this approach as currently implemented.

**Results:**

In this proof-of-principle study, we analyze 127 CRMs and focus on four TFs that control transcription of target genes along the anterio-posterior axis of the embryo early in development. For all four of these TFs, there is some degree of conserved flanking sequence that extends beyond the predicted binding regions. A potential role for these conserved flanking sequences may be to enhance the specificity of TF binding, as the abundance of these sequences is greatly diminished when we examine only predicted high-affinity binding sites.

**Conclusions:**

Expanding PWMs to include sequence context-dependence will increase the information content in PWMs and facilitate a more efficient functional identification and dissection of CRMs.

## Background

The control of gene expression during development in *Drosophila* and other metazoans is tightly directed by *cis-*acting regulatory sequences in the genome. These DNA sequences modulate expression of target genes by binding protein transcription factors (TFs) [1]. Contact between a TF and DNA sequence is mediated through the TF’s DNA binding domain(s) in a sequence dependent manner [2-4]. Each TF has one or more of a variety of different DNA binding domains, including zinc fingers and homeoboxes [5-9]. Significant efforts have been undertaken to comprehend the organization of DNA sequence at known binding regions and further understand how this influences the ability of a TF to bind.

**Figure 1:**
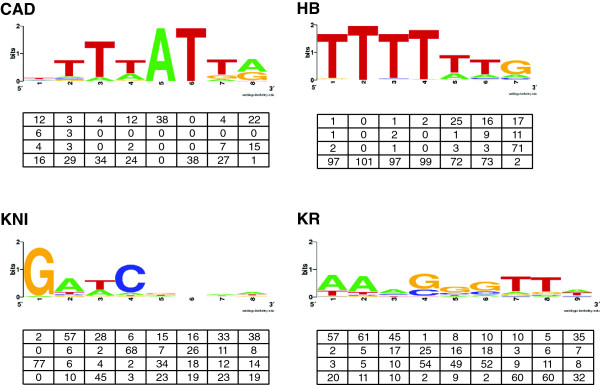
**Transcription factor binding sequences and position weight matrices.** TF consensus binding site sequences for CAUDAL (CAD), HUNCHBACK (HB), KNIRPS (KNI) and KRUPPEL (KR) are shown above the PWM generated from experimentally-verified TF binding regions [30,31]. The height of each of the nucleotide bases reflects the relative likelihood of their presence at that position in the TF binding region.

Our understanding of TF-DNA interactions has been greatly aided by bioinformatic tools developed to analyze DNA sequences obtained from experimental studies focused on identifying TF binding regions. A key approach involves the construction of a position weight matrix (PWM) [10-15]. In PWM-based models, known binding regions for a given TF are first characterized by utilizing experimental data from DNA footprinting assays, yeast one-hybrid assays, chromatin immunoprecipitation-sequencing (ChIP-seq) or protein binding microarrays (PBMs) [2,16,17]. The binding regions are then aligned and trimmed to some minimal sequence length, *L*, and the frequency at which each nucleotide is observed at each position is recorded in a matrix of dimension 4 × *L*[18] (Figure 1). Once a PWM is constructed, these models aid in the discovery of *de novo* binding sites *in silico*, providing predictions for the location of additional binding regions in the genome, without the need for technically challenging *in vitro* binding assays [10,14].

A host of computational tools have been developed that seek to streamline the discovery of *de novo* TF binding regions using PWMs [10,14,19,20]. However, a major limitation of PWMs is their potential to lose information content during construction. The lengths of PWMs are often determined based on an optimal alignment between minimal sequences of varying length, potentially eliminating bordering regions crucial to determining a TF’s binding preference [21-23]. Extending PWMs may therefore serve to increase their information content, and thus their predictive power [24]. One limitation resulting from the experimental approaches to isolate TF-bound DNA fragments [25,26], is that there may be additional, but non-contiguous, bases that are fundamentally important to TF binding initiation (or transient TF-DNA binding) which are not represented in the experimental data and therefore not taken into account during traditional PWM construction. A potential explanation for this lack of information content in canonical PWM construction is the omission of secondary binding by TFs with multiple DNA binding domains [2]. For example, in *Drosophila* the HUNCHBACK TF has two distinct C_2_H_2_-type zinc-finger binding domains [9]. If multiple DNA binding domains contact sequences separately then each domain may contribute to the overall binding of the TF. Accordingly, in the case where there are two binding domains, one of the DNA binding regions may be either: a) discarded because it fails to meet minimal fragment size requirements or b) incorporated into a combined alignment along with the sequences representing regions bound by the other binding domain. Either of these scenarios may lower the information content of the PWM. The first scenario may result in a PWM that does not include all nucleotides necessary for *in vivo* binding (i.e. a PWM representing the actual binding region may be longer than that which is constructed from the current experimental data). The second, on the other hand, points to an even larger problem in PWM construction: the possibility that a TF may have two different modes of binding, and thus a single unique PWM is insufficient to predict all DNA binding regions, which there has been strong evidence to support in the case of mammalian DNA binding proteins [2].

To address the limitations of PWMs, we align and analyze predicted binding regions for four well-studied TFs in 127 *cis*-regulatory modules (CRMs) that are essential to direct gene expression along the anterio-posterior axis in early *Drosophila* development. Our analysis indicates that the current PWMs for all four TFs examined exclude significant biases towards a given base, or bases, in specific positions in the neighboring sequences and that the information content of these PWMs can be improved by including these additional sequences.

## Methods

### *Cis*-regulatory module and flanking sequences

**Figure 2:**
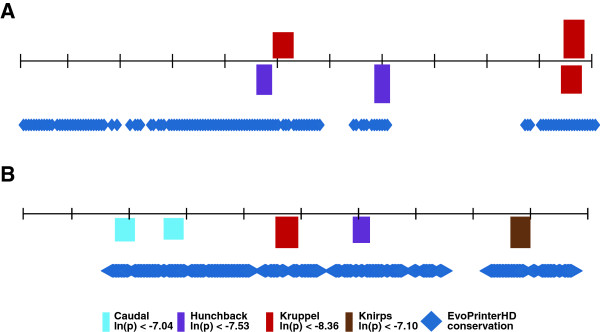
**Conserved sequence beyond known TF binding regions.** PATSER predicted binding regions for CAD, HB, KNI and KR are shown above EvoPrinterHD conservation predictions (see Methods for details) for sub-sections of the **A)** Even-Skipped Stripe 5 CRM (bases 276 to 494) and **B)** Paired Stripe 2P CRM (bases 800 to 1000). The height of each rectangle indicating a predicted binding region is proportional to its strength.

We identify 114 genes of interest that display a pattern of differential expression along the anterio-posterior axis during *Drosophila* development at or before stage 5 (all genes and expression data from FlyBase) [27]. In order to compile a database of CRMs, we utilize the REDfly database [28] to search each of the 114 genes and identify all those with *in situ-*verified CRMs (47 genes, consisting of 127 CRMs). Since we are investigating the flanking region (possibly up to 100 bp) of each PWM-predicted TF binding region, and TF binding sites may be predicted within the first or last 100 bps of a given CRM, for each CRM we obtained the entire sequence of the CRM and 100 bp of flanking sequence in each direction. The DNA sequences of both the original CRMs and these extended CRMs are available in the Additional file 1: Dataset S1 and Additional file 2: Dataset S2). To analyze sequence conservation in each CRM we run EvoPrinterHD strict [29] with default settings, specifying four *Drosophilid* species for comparison: *D. pseudoobscura, D. ananassae, D. erecta, and D. sechellia* (results shown in Figure 2).

### Bioinformatic analysis

The PWMs we use for CAUDAL (CAD) [30], HUNCHBACK (HB), KNIRPS (KNI) and KRUPPEL (KR) [31] are as previously described. For our analysis, we run PATSER [14] with default settings (i.e.: the total number of pseudo-counts is set to 1 and the background sequence A/T content is 0.3 and C/G content is 0.2). To determine score (ln(p-value)) cutoffs, we first observe the distribution of scores PATSER assigns, using each of the four individual PWMs, to all the known binding regions used to construct each of the original respective PWMs. The cutoffs used are calculated by taking the 75^th^ and 50^th^ percentile cutoff of all these scores, and are referred to as 'strong’ and 'weak’ cutoffs respectively. We then run PATSER on each of the original 127 CRMs (excluding flanking regions) with each of the four PWMs to predict binding regions. Only those regions scoring above the respective cutoff are used for further analysis and are referred to as core PWM predicted binding regions (PWM-PBRs). Note that scores are all negative, so 'scoring above’ refers to PATSER outputting a ln(p-value) less than or equal to the cutoff. The strong cutoff is more stringent, only predicting binding regions that receive a score less than or equal to that obtained from the top 25% of known binding region scores (108 CAD, 157 HB, 79 KNI, and 18 KR sites), representing binding regions that are most similar to the consensus core binding region for the given TF. The weak cutoff is less stringent, predicting binding regions that receive a score less than or equal to that obtained from the top 50% of known binding region scores (430 CAD, 450 HB, 359 KNI, and 127 KR sites), representing binding regions that are contained in a larger range of similarity to the consensus core binding region for the given TF. One should note that these cutoff scores are TF-specific and are different for each of the four TFs analyzed. In cases where overlapping binding regions were identified, both regions are included in all subsequent analyses. Lists of all the known binding regions for each TF, their corresponding ln(p-value) obtained using PATSER, and whether they fall into the 75^th^ percentile, 50^th^ percentile, or neither are available in the Additional file 3: Table S1.

### Statistical analysis

**Figure 3:**
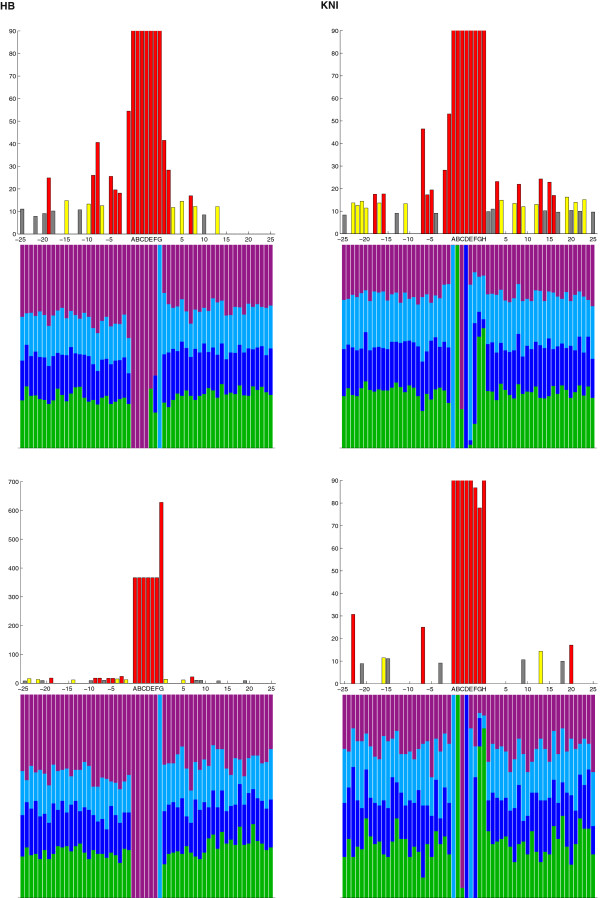
**Sequence conservation surrounding HB and KNI DNA binding sites.** Graphs on the left and right sides correspond to results from HB and KNI respectively. In each case, the top pair of graphs were generated from sites filtered by the weak ln(P) cutoff score and the bottom graphs by the strong ln(P) cutoff score (see Methods for details). The x-axis of each graph is the position relative to the consensus binding site, with nucleotides in the core binding site marked with letters and the neighboring 25 bp on each side marked numerically by their position relative to the boundaries of the core binding region. In each upper graph, the y-axis indicates the Chi-squared value from a test in which the null hypothesis is an expected ratio at each nucleotide position of T (0.3), A (0.3), C (0.2), and G (0.2). Chi-squared values shown have been capped at 90 for clarity. Colored bars indicate statistical significance and are based on the lower-bounds of the α-value (Chi-squared values) for 0.05 (7.815, gray), 0.01 (11.345, yellow) and 0.001 (16.266, red). For example, the HB binding region statistically significantly varies from the genome-wide nucleotide distribution one and two bps downstream of the core binding site in the weak cutoff graph, but not in the strong cutoff graph. Each lower graph depicts the frequency of A (green), C (dark blue), G (light blue), and T (purple) at each position.

**Figure 4:**
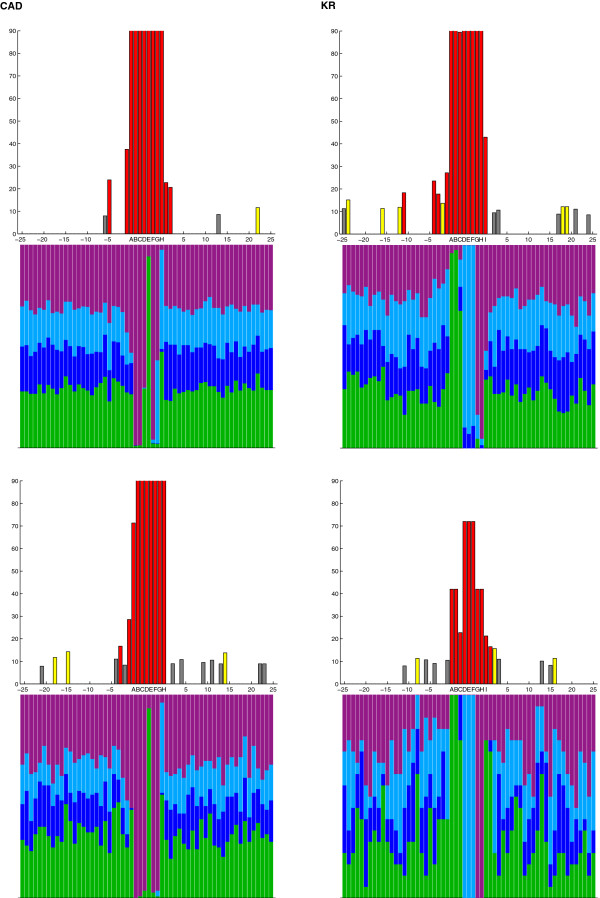
**Sequence conservation surrounding CAD and KR DNA binding sites.** Graphs on the left and right sides correspond to results from CAD and KR respectively. In each case, the top pair of graphs were generated from sites filtered by the weak ln(P) cutoff score and the bottom graphs by the strong ln(P) cutoff score (see Methods for details). The x-axis of each graph is the position relative to the consensus binding site, with nucleotides in the core binding site marked with letters and the neighboring 25 bp on each side marked numerically by their position relative to the boundaries of the core binding region. In each upper graph, the y-axis indicates the Chi-squared value from a test in which the null hypothesis is a expected ratio at each nucleotide position of T (0.3), A (0.3), C (0.2), and G (0.2). Chi-squared values shown have been capped at 90 for clarity. Colored bars indicate statistical significance and are based on the lower-bounds of the α-value (Chi-squared values) for 0.05 (7.815, gray), 0.01 (11.345, yellow) and 0.001 (16.266, red). Each lower graph depicts the frequency of A (green), C (dark blue), G (light blue), and T (purple) at each position.

For each TF we aggregate the core PWM-PBRs predicted by PATSER along with a fixed number, *n,* of bps of flanking sequence on each side, obtained from the 127 extended CRMs with the core PWM-PBR in the center and *n* bps on each side. When binding sites are found on the reverse complement strand, we use the reverse complement sequence for analysis. Since each core PWM-PBR is exactly the same length (*L*) as the PWM associated with the given TF, each sequence of DNA in the list is the same length. This allows us to compute the frequency of each base (A/C/G/T) at each position (from –*n* to *L* + *n*). These frequencies are shown for *n* = 25 (50 total bases beyond the *L* bases in the original PWM) in the case of both strong and weak cutoff scores in Figures 3 and 4 (bottom bars on each graph) and are listed in the Additional file 4: Table S2.

For each TF and cutoff score, given the list of PBRs (including flanking regions on both sides of the core PWM-PBRs), we run a Chi-squared test on each position with the null hypothesis that at any given location, the distribution of A/C/G/T is exactly the same as the genome-wide distribution [32], A(0.3):C(0.2):G(0.2):T(0.3). We note here that the overall nucleotide frequency in the 127 extended CRMs, A(0.2845):C(0.2155):G(0.2155):T(0.2845) is not significantly different from the genome-wide distribution (*χ*^2^ test, p-value > 0.95). We analyze the results of these tests at three different confidence levels α = 0.05, α = 0.01 and α = 0.001. We choose more than one confidence level to control the familywise error rate for multiple comparisons. A simple Bonferroni correction leads to a corrected alpha value obtained by dividing alpha by the number of Chi-squared tests (ie: in the *n = 25* case, α = 0.05/50 = 0.001 is the Bonferroni corrected value corresponding to α = 0.05). Thus, although the three alpha values stated can be interpreted without a Bonferroni correction, α = 0.001 can also be interpreted as a Bonferonni corrected alpha corresponding to α = 0.05. The Chi-squared values obtained are shown for *n* = 25 in Figures 3 and 4 (top bars on each graph) and actual values for each nucleotide are in the Additional file 4: Table S2. Note that in Figures 3 and 4, the color-coding corresponds to the smallest alpha value of those analyzed in which the null hypothesis is rejected.

### Software availability

The web application that is used to run this analysis is freely available for non-commercial use at: http://drewell.sites.hmc.edu/projects/sequence_context_grapher.html.

### JASPAR database search

Using the alignment produced by the bioinformatic analysis for the 'weak’ (50^th^ percentile) cutoff described above, a single PWM was constructed from a portion of the flanking sequence of HB showing statistically significant nucleotide bias (-9 to -1 relative to the core PWM-PBR) for use in a JASPAR alignment search [33]. The top 5 *Drosophila melanogaster* TFs that give similarity scores to this PWM (similarity scores > 86%) are manually annotated for expression pattern in early (stages 1-5) embryos [34].

### Testing predictions for expanded HB PWMs

When expanding the HB PWM, we use a 2-step process. First, we choose an initial cutoff score and predict and align all binding sites using the original core PWM as described in 'Bioinformatic Analysis’. The PWM-PBRs identified with the original core PWM using this initial cutoff score are then extended by including flanking regions of interest (ranging from -25 to +25). An extended PWM is constructed from the base frequencies of these extended PWM-PBRs. Next, PATSER is used with the extended PWM to determine the score for each of the extended PWM-PBRs constructed from the set of predicted HB binding sites with the initial cutoff score. Computing the percentiles of these scores, in the same way as is described for the original PWM analysis in 'Bioinformatic Analysis’, we obtain a secondary cutoff score. Lists of the extended PWM-PBRs, their corresponding ln(p-value) obtained using PATSER with the corresponding extended HB PWM, and whether they fall into the 0^th^, 25^th^, 50^th^ or 75^th^ percentile are available in the Additional file 5: Table S3. We run Patser again with the extended PWM and secondary cutoff score on the IAB7b CRM, which contains one known functional HB binding site [35]. This allows us to then compare the location of the predicted binding sites obtained using extended HB PWMs of varying lengths to the known HB binding site to determine which PWMs result in the lowest number of false positive and false negative predictions.

### Analyzing expanded PWMs using ChIP-seq datasets

To analyze the predictive power of the extended PWMs, we first choose initial cutoff scores representing the 25^th^ and 50^th^ percentile scores and generate PWM-PBRs for each of the four TFs as described in 'Bioinformatic Analysis’. We then extend those core PWM-PBRs (core) to include the core and all highly significant (*χ*^2^ test, α < 0.001) flanking sequence context-dependent biases (extended), as well as the core with -25 to +25 flanking regions used to generate the PWM-PBR (full). We use a secondary cutoff score corresponding to the 0^th^ percentile as described in 'Testing Predictions for Expanded HB PWMs’. ChIP-seq datasets for each TF from the BDGP [36] are filtered to include only those peaks with more than 100 bp of sequence. We run PATSER with each of the three different PWMs for each TF on their respective TF peaks and score a true positive prediction when the PWM predicts at least one TF binding site. For each ChIP-seq peak, we calculate the nucleotide distribution within the peak and create 10 'scrambled peaks’, random DNA sequences of the same length and nucleotide distribution. We then run PATSER with each of the three different PWMs for each TF on these scrambled peaks and score a false positive prediction when the PWM predicts at least one TF binding site. Both the true positive and false positive results for the 25^th^ and 50^th^ percentile initial cutoff scores at the 0^th^, 25^th^, 50^th^, and 75^th^ percentile secondary cutoff scores are available in the Additional file 6: Table S4.

## Results and discussion

When considering the possibility of sequence context-dependence for TF binding, evidence has pointed toward the existence of nucleotide biases at positions in close proximity to a region experimentally verified or computationally predicted to bind a TF [5,37]. To test this idea, we analyze 127 CRMs that are active during early *Drosophila* development for predicted binding regions for four TFs using PATSER (see Methods for details). These four TFs: CAUDAL (CAD), HUNCHBACK (HB), KNIRPS (KNI) and KRUPPEL (KR) are all critical for normal development and are present in spatially restricted patterns along the anterio-posterior axis in early embryogenesis [38]. A number of *in vivo* confirmed minimal binding regions have been characterized for these TFs and the existing PWMs for each of these factors range in size from 7 to 9 bp (Figure 1) [30,31]. Of greatest importance for this study, their current canonical PWMs have been proven to have greater predictive power for experimentally validated TF binding regions, when compared to other published PWMs [31]. If context-dependent biases are present in sequences near these characterized binding regions, we predict that these bases would be evolutionarily conserved. By combining PATSER [14] analysis with EvoPrinterHD [29] analysis, we are able to identify several examples of extended regions of sequence conservation surrounding evolutionarily conserved TF binding regions, including portions of the *even-skipped* stripe 5 CRM and the *paired* stripe 2 CRM (Figure 2). In all cases within the depicted portions of the *even-skipped* stripe 5 CRM and *paired* stripe 2 CRM, predicted TF binding regions are flanked by substantial extended sequence conservation on one or both sides. This presents a testable hypothesis: that these regions of extended conservation contain functionally important flanking bases that are important for robust TF binding.

To address the hypothesis that there may be sequence context-dependent binding for the four TFs, we investigate the sequences 25 bp up- and downstream of defined core PWM-predicted binding regions (PWM-PBRs) (described in detail in Methods). Alignment of the core PWM-PBRs and their flanking regions for each individual TF does indeed reveal a statistically significant enrichment of certain bases outside of the core PWM-PBRs (Chi-squared test, α < 0.05). A very clear example of this enrichment with high statistical significance (Chi-squared test, α = 0.001) is found at binding regions for HB. Using the weak cutoff value (see Methods for details), beyond the HB core PWM-PBR there is context-dependent bias at the first two and the 7th nucleotide downstream (+1, +2 and +7) of the core PWM-PBR (Figure 3). In addition, there are four clusters of context-dependent bias upstream of the HB core PWM-PBR at positions -1, -3 to -5, -8 to -9 and -19 (Figure 3). KNI seems to follow a similar pattern to HB, with nucleotide enrichment bias at 5 positions downstream and 7 upstream of the core PWM-PBR (Figure 3). This enrichment bias is also seen for CAD and KR (Figure 4), but is not as prevalent. CAD and KR display only short stretches of sequence with robust context-dependent bias, and in both cases these sequences are largely contiguous to the core PWM-PBRs (Figure 4). For all four TFs the enrichment biases at positions neighboring the defined core PWM-PBRs could be incorporated in to expanded PWMs.

**Figure 5:**
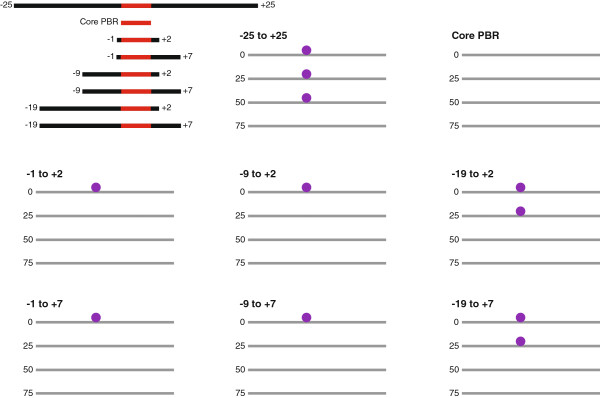
**High stringency HB binding site predictions in the IAB7b CRM.** PWMs corresponding to the 7 bp core predicted binding region (PBR, red) or the core PBR with flanking sequences of various sizes (black) were used to predict HB binding sites in the 828 bp IAB7b enhancer with an initial cutoff threshold ln(p-value) = -7.53 (corresponding to the 50^th^ percentile). For each individual PWM, a secondary cutoff at the 0^th^, 25^th^, 50^th^ or 75^th^ percentile of all the ranked sites (see Methods for details) was also applied. Note that at this primary cutoff score the core PBR fails to predict the true positive HB binding site (purple circle) and as the length of the PWM is increased more robust predictions are made.

**Figure 6:**
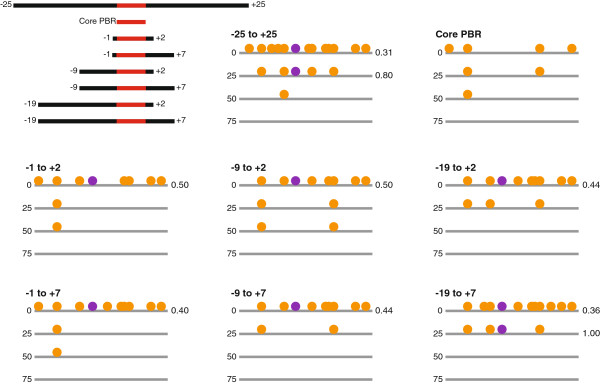
**Low stringency HB binding site predictions in the IAB7b CRM.** PWMs corresponding to the 7 bp core predicted binding region (PBR, red) or the core PBR with flanking sequences of various sizes (black) were used to predict HB binding sites in the 828 bp IAB7b enhancer with an initial cutoff threshold ln(p-value) = -6.77 (corresponding to the 25^th^ percentile). For each individual PWM, a secondary cutoff at the 0^th^, 25^th^, 50^th^ or 75^th^ percentile of all the ranked sites (see Methods for details) was also applied. At this lower stringency primary cutoff score there are many more false positive binding sites predicted (orange circles). The core PBR fails to predict the true positive HB binding site (purple circle). The most robust predictions are made with the -19 to +7 PWM at the 25^th^ percentile secondary cutoff, which predicts the true positive HB site and just three false positive sites (normalized value = 1.00, the relative performance of all other PWMs that predict the true positive HB site is measured against this value).

To directly test the ability of expanded PWMs to accurately predict TF binding sites, we examine the performance of multiple HB PWMs of varying length on the IAB7b CRM from the bithorax complex. This 828 bp CRM has been extensively characterized [35,39,40] and contains a single HB binding site in the highly-conserved 154 bp signature motif [35], which is sufficient to account for the functional repression of the CRM mediated by HB in the anterior half of the embryo. Using a relatively stringent initial cutoff (equivalent to the 50^th^ percentile, see Methods for details), the 7 bp HB PWM corresponding to the core PWM-PBR fails to predict any binding sites in the enhancer at all secondary cutoff values (Figure 5, see Methods for details). Extending the PWM by only 3 bp (from -1 to +2 relative to the core PBR) allows the prediction of the true positive site (Figure 5). As the PWM is expanded to include more flanking sequence it continues to perform better. The most robust prediction of the true positive HB binding site is with the -25 to +25 PWM. At this stringent initial cutoff value there are no false positive predictions with any of the HB PWMs. We also investigate the predictive power of the PWMs under less stringent conditions, utilizing an initial cutoff equivalent to the 25^th^ percentile (see Methods for details). Using this less stringent initial cutoff there are many more predicted false positive HB binding sites (Figure 6). The HB PWM corresponding to the core PWM-PBR gives the highest false negative rate; it fails to predict the single true positive HB site in the enhancer at any of the secondary cutoff values (Figure 6). Once more, as the PWM is extended it generally performs better. The most robust true positive predictions are with the -19 to +7 HB PWM (Figure 6), suggesting that expanding the PWM to include all flanking sequences that show a statistically significant nucleotide bias improves the predictive power of the PWM. With the less stringent initial cutoff value, in all cases, shorter HB PWMs reduce the false positive rate but at the expense of increasing the false negative rate (Figure 6).

**Figure 7:**
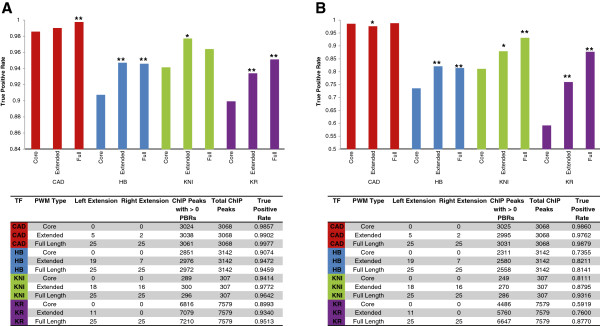
**Position Weight Matrix extension enables high fidelity identification of Predicted Binding Regions in ChIP-seq peaks.** Three different PWMs were constructed for CAD (red), HB (blue), KNI (green), and KR (purple) respectively, corresponding to; the core PWM-PBR (core), a region encompassing the core PBR and all statistically significant flanking sequence context-dependent biases (extended), and the core with 25 bp of flanking sequence on each side (full). For each individual PWM, PATSER was run on filtered ChIP-seq peaks using a low stringency (**A**, corresponding to the 25th percentile) or high stringency (**B**, corresponding to the 50th percentile) primary cutoff and then a secondary cutoff score corresponding to the 0th percentile (see Methods for full details). Charts depict the true positive rate (defined as the number of ChIP-seq peaks for which the PWM predicts > 0 binding regions divided by the total number of ChIP-seq peaks). Statistical significance of divergence in true positive rate of expanded PWMs from the corresponding core type PWM is indicated with asterisks (Two-tailed Fisher exact test, * denotes P-value < 0.05, ** denotes P-value < 0.0001).

To further analyze the performance of the extended PWMs, we generate three different PWMs for each of the four TFs and test the predictive power on ChIP-seq peak sequences for each TF. The three different PWMs correspond to: the core PWM-PBR (core), a region encompassing the core PBR and all statistically significant flanking sequence context-dependent biases (extended), and the core with 25 bp of flanking sequence on each side (full). Comparison of the ability of each of the three PWMs to correctly identify at least one binding site in individual ChIP-seq peaks for each TF reveals that in each case, with the exception of the CAD PWM at low stringency (see Methods for details), the extended PWMs significantly outperform the core PWMs (Figure 7).

**Figure 8:**
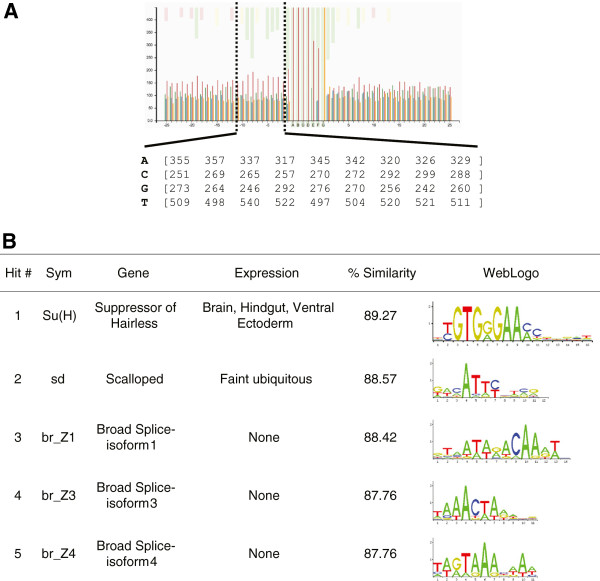
**Predicted transcription factor binding sites in sequence adjacent to the core HB binding region. A**. HB has a region of context conservation extending from the core PBR downstream from the -1 to -9 base positions. Using the weak ln(P) cutoff score alignment, a PWM was generated of this region and subjected to a JASPAR alignment search (see Methods for details). **B**. The top 5 hits are shown with WebLogo representations of their PWMs. Of the 5, only su(H) and sd are expressed in the early embryo, with organ specific and weak ubiquitous expression respectively.

There are many potential reasons for the fact that all four TFs exhibit context-dependent biases within their extended binding regions, including specific interactions dependent on the physical constraints of the TF contacting DNA, nucleosomes or co-factors. To address the possibility that co-factors may in fact be recruited to these neighboring sequences we investigate a cluster of context-dependent sequence exhibiting strong statistical significance (the -9 to -1 bp region relative to the HB core PBR, Figure 8a) for additional TF binding sites. Alignment of the PWM corresponding to the -9 to -1 DNA region to all PWMs available in the JASPAR database identifies putative binding sites for other *Drosophila* TFs (see Methods for details). Of the top five binding sites, ranked according to their binding motif’s similarity to the PWM constructed from the -9 to -1 HB region, only two (*su(H)* and *sd*) correspond to TFs expressed in the early embryo (Figure 8b). None of the top five ranked TFs are known to have any functional interaction with HB, suggesting that secondary sequence context-dependence is not sufficiently explained by co-factor binding alone.

A further testable explanation for the fact that all four TFs exhibit context-dependent biases at their extended binding regions may be that these TFs have multiple DNA binding domains, each of which contacts different nucleotides independently [2], but together act to increase the TF’s binding affinity for target sequences. If this is the case, these secondary DNA binding domains may enhance, but not replace the function of the primary, canonical binding domain in the TF protein. To assess this hypothesis, we compare the binding regions predicted by PATSER using both strong and weak cutoff scores (see Methods). While many of the nucleotide position biases persist at either cutoff score, we find a general decrease in the number and significance of context-dependent biases for all four TFs when we only consider strong sites (Figures 3 and 4 and Additional file 4: Table S2). For example, context-dependent biases within 15 bp of the KNI PWM-PBR are greatly reduced when we only consider strong sites - of the 12 enriched biases found with the weak cutoff, only one remains (Figure 3). The range of decrease across the four TFs is variable (Figures 3 and 4). Overall, these results suggest two separate binding regimes: (1) if a TF has secondary, non-contiguous binding, stronger core binding regions may overcome a paucity of context-dependent biases in the flanking sequences, whereas weaker core binding regions may depend more heavily on these biases; (2) if a TF exhibits contiguous biases, these biases may simply suggest a larger canonical core binding region. However, in some cases, there are nucleotides that are found to be significant only when the strong cutoff score is used. For example, two new biases are detected at +20 and -23 relative to the KNI PWM-PBR (Figure 3). This finding supports a hypothesis that strong and weak core binding regions may in fact have two different functional roles that allow the TF to bind in a different mode, again posing the question of whether it is valid or not to represent a TF binding with one unique PWM [2].

**Figure 9:**
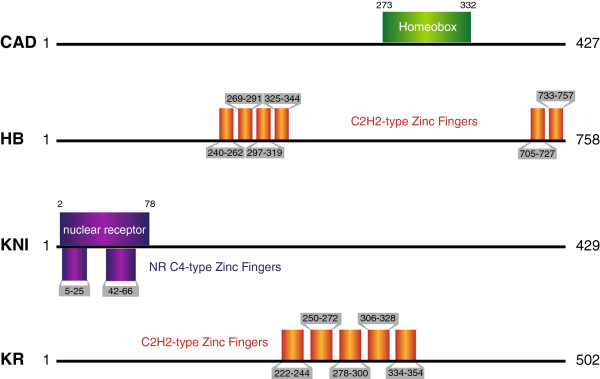
**DNA-binding domains in CAD, HB, KNI and KR transcription factors.** CAD has a single homeobox domain (green), HB and KR have multiple C_2_H_2_-type zinc-finger domains (orange), and KNI has two nuclear receptor C_4_-type zinc fingers (purple).

To address the possibility that the presence of secondary sequence context-dependence in the case of a particular TF is due to the TF having multiple binding domains, as previously shown for a large number of mammalian TFs [2], we investigate the binding domains of each of the four *Drosophila* TFs. HB has two groups of C_2_H_2_-type zinc fingers, separated by over 350 amino acids, while KR has only one group of fingers (Figure 9). This may explain the stark difference between the significance profiles at flanking sequences neighboring defined PWM-PBRs for HB and KR (Figures 3 and 4). In addition, it may reveal a relationship between the predicted binding strength and number of context-dependent biases for each TF. One model is that the group of four zinc-fingers in the center of HB form the core DNA-binding domain (capable of binding to the PWM-PBR), while the other group of two auxiliary zinc-fingers form a secondary binding domain (capable of binding neighboring sequences) with less contribution to the overall binding stability (Figure 9). There is some experimental evidence to support this model, as the highly conserved multi-zinc finger TF CTCF has been shown to contact DNA nucleotides at least 12 bp (and possibly up to 40 bp) apart at the human c-myc promoter [41]. In comparison, the single group of zinc-fingers in KR form a single DNA binding domain which can only bind the core PWM-PBR and directly adjacent sequences (Figure 9). CAD also has only one binding domain (a homeobox), potentially explaining CAD's lower level of secondary sequence context-dependence, when compared to HB or KNI. Of the four TFs, KNI is the most puzzling, exhibiting secondary sequence context-dependency while having only a single annotated binding domain (Figure 9). One potential explanation may be that, unlike in the case of KR where the two zinc fingers are only six amino acids apart, the two C_4_-type zinc fingers in KNI are separated by 17 amino acids. This increased separation may in fact allow the two zinc fingers to act as functionally distinct DNA binding domains, as is the case for HB.

## Conclusions

Taken together these data suggest that current PWMs may not be optimal to explain the complexity of TF binding. Although we only test four TFs in this study, we demonstrate that all four TFs exhibit context-dependent biases towards given nucleotides both contiguously with the defined minimal binding region and non-contiguously in flanking DNA sequences, thus providing a foundation for this to be explored more broadly. An additional intriguing question for future study will be to investigate if the context-dependent bias persists at predicted TF binding regions in other genomic regions that are not characterized as CRMs. By taking these secondary context-dependencies into account, we propose that the information content of PWMs can be expanded in many cases. This expansion would not only provide better predictions of true TF binding regions in the genome, but may also help improve estimates of relative binding affinities at specific sites, allowing one to understand the molecular basis for the difference between weak and strong binding sites. The ability to identify novel CRMs and decipher the sequence organization at CRMs relies heavily on a concrete understanding of TF binding preferences. Improving the information content of PWMs and our comprehension of TF binding events will contribute to these continued efforts.

### Availability of supporting data

The data sets supporting the results of this article are included within the article (and its Additional file 3: Table S1, Additional file 4: Table S2 and Additional file 5: Table S3, Additional file 1: Datasets S1 and Additional file 2: Datasets S2, and Supporting legends.)

## Competing interests

The authors declare that they have no competing interests.

## Authors’ contributions

JLS created the web application, ran all bioinformatic and statistical analyses, and tested expanded HB PWMs. ASB compiled the 127 CRMs, ran the EvoPrinterHD analysis, performed the JASPAR database search, and analyzed each TF’s binding domain(s). The manuscript was written by JLS, ASB, RAD and JMD, and the overall project was conceived and guided by RAD and JMD. All authors read and approved the final manuscript.

## Supplementary Material

Additional file 1 **Dataset S1.** DNA sequences of the original CRM (Dataset S1). The original CRM folder (Dataset S1) contains the 127 FASTA files for the CRMs we used. Click here for file

Additional file 2 **Dataset S2.** DNA sequences of the extended CRMs (Dataset S2). The extended CRM folder (Dataset S2) contains those same 127 CRMs, extended by including the neighboring 100 bp both up- and downstream of the CRM. The extended CRM FASTA filenames are identical to the original, but with a ' + ’ appended to the filename (i.e., the original Kr_4.fasta file in File S1 has a corresponding Kr_4 + .fasta file in File S2). Click here for file

Additional file 3: Table S1 PATSER scores for predicted binding regions. Each sheet contains a list of the sequences in predicted binding regions for a particular TF. Sequences are ordered by their ln(p‒value), as computed by PATSER (see Methods for details). The scores highlighted in yellow indicate those scoring at or above (with ln(p‒value) less than or equal to) those included in the 75^th^ percentile (strong cutoff sites). The scores highlighted in green indicate those scoring at or above (with ln(p‒value) less than or equal to) those included in the 50^th^ percentile (weak cutoff). Recall that the 75^th^ and 50^th^ percentile cutoff scores are specific for each TF and thus vary from sheet to sheet. Click here for file

Additional file 4: Table S2 Nucleotide frequencies in flanking sequences neighboring predicted binding regions. Each sheet corresponds to a particular TF and cutoff percentile (labeled as weak or strong, see Methods). The column labeled 'position’ assigns alphabetic values to the consensus core PWM-predicted binding regions (PWM‒PBRs) and numeric values to the neighboring flanking sequences, ranging from ‒25 to ‒1 upstream and from 1 to 25 downstream. The next four columns, labeled 'A’, 'C’, 'G’, and 'T’, contain the frequency observed for each nucleotide at the specified position when all PWM-PBRs are considered for that particular TF and cutoff percentile. The column labeled 'chi‒squared’ contains the corresponding chi-squared value for each position. Click here for file

Additional file 5: Table S3 PATSER scores for extended PWM-PBRs. Each sheet contains a list of the sequences in the extended PWM-PBRs for a particular initial cutoff score and length of flanking region. Sequences are ordered by their ln(p-value), as computed by PATSER using the corresponding extended HB PWM (see Methods for details). The scores highlighted in yellow indicate those scoring at or above (with ln(p-value) less than or equal to) those included in the 75^th^ percentile (strongest cutoff sites). The scores highlighted in green indicate those scoring at or above (with ln(p-value) less than or equal to) those included in the 50^th^ percentile (strong cutoff sites). The scores highlighted in orange indicate those scoring at or above (with ln(p-value) less than or equal to) those included in the 25^th^ percentile (weak cutoff sites). The scores highlighted in blue indicate those scoring at or above (with ln(p-value) less than or equal to) those included in the 0^th^ percentile (weakest cutoff sites). This includes all sites detected with the initial cutoff score. Recall that the 0^th^, 25^th^, 50^th^ and 75^th^ percentile cutoff scores are specific for each initial cutoff score and length of flanking region and thus vary from sheet to sheet. Click here for file

Additional file 6: Table S4 True and false positive results for extended PWM-PBRs on ChIP-seq peaks. Each sheet corresponds to a particular TF. The name of the TF, the total number of ChIP-seq peaks, and the number of scrambled peaks used for the analysis are listed in the first column. In the other columns, each row corresponds to a PATSER run for the given TF, ChIP-seq peaks and scrambled peaks. The primary and secondary cutoff percentiles used, as well as the number of nucleotides extended to the left (upstream) and right (downstream) of the core PWM-PBR are listed. The results of each run are shown as the number of ChIP peaks containing at least one predicted binding site, which is used to calculate the true positive rate, and the number of scrambled ChIP peaks containing at least one predicted binding site, which is used to calculate the false positive rate. The last column contains the ratio of true positive to false positive. To highlight settings in which the sensitivity increased in at least one of the extended PWM-PBRs compared to the core, we have highlighted those true positive rates in green, to highlight settings in which the specificity increased in at least one of the extended PWM-PBRs compared to the core, we have highlighted those false positive rates in pink, and to highlight settings in which the ratio of true to false positives increased in at least one of the extended PWM-PBRs compared to the core, we have highlighted those ratios in yellow. Click here for file
